# Structure Determination of a Single Bioapatite Crystal from an Archeological Metastatic Cancer Bone – a TEM Case Study

**DOI:** 10.1021/acsomega.6c05043

**Published:** 2026-10-12

**Authors:** Zsolt Dallos, Marco Santucci, Krisztián Kiss, Melanie Bauer, Michael Burtscher, Zoltán Kovács, Tamás Szeniczey, Antónia Marcsik, Erika Molnár, Krisztina Buczkó, Daniel Kiener, Ute Kolb, Tamás Hajdu, István Dódony

**Affiliations:** † Institute of Geoscience, 9378 Friedrich Schiller University Jena, Jena D-07743, Germany; ‡ Institute of Inorganic Chemistry and Analytical Chemistry, 9182 Johannes-Gutenberg-University, Mainz, Mainz D-55128, Germany; § Department of Biological Anthropology, Eötvös Loránd University, Budapest H-1117, Hungary; ∥ Institute of Archaeogenomics, ELTE Research Centre for the Humanities, Budapest H-1097, Hungary; ⊥ Institute of Practical Methodology and Diagnostics, Faculty of Health Sciences, University of Miskolc, Miskolc H-3515, Hungary; # Lendület “Momentum” Bioarchaeology Research Group, MTA−ELTE HTK, Budapest H-1097, Hungary; ∇ Department Materials Science, 27268 Technical University of Leoben, Leoben A-8700, Austria; ○ HUN-REN Centre for Energy Research, Budapest H-1121, Hungary; ◆ Department of Biological Anthropology, University of Szeged, Szeged H-6726, Hungary; ¶ Center for Natural Sciences, Limnology Research Group, University of Pannonia, Veszprém H-8200, Hungary; ▶ Department of Botany, Budapest, Hungarian National Museum Public Collection Centre, Hungarian Natural History Museum, Budapest H-1431, Hungary; ▷ Institute for Applied Geosciences, Technical University of Darmstadt, Darmstadt D-64287, Germany; ◀ Centre for Applied Bioanthropology, Institute for Anthropological Research, Zagreb 07743, Croatia; ◁ NANOLAB, Research Institute of Biomolecular and Chemical Engineering, University of Pannonia, Veszprém H-8201, Hungary

## Abstract

The inorganic component of bone, bioapatite, is generally regarded as a poorly crystalline, carbonate-substituted hydroxyapatite. However, a direct structural and chemical characterization of bioapatite has not yet been achieved due to the extremely small size of its crystals — typically only a few tens of nanometers — and their intimate association with the organic matrix of bone. In this study, we examined a single crystal of bioapatite extracted from an archeological, metastatic bone cancer sample using 3D ED (3-Dimensional Electron Diffraction), which enabled structural analysis of the three-dimensional reciprocal space despite the nanometric crystal dimensions. Conventional structural techniques, such as single-crystal X-ray diffraction, require crystals several orders of magnitude larger than those found in bone, rendering them inapplicable to individual bioapatite crystallites. Powder-based methods, on the other hand, can only provide averaged structural information across billions of crystals, obscuring the intrinsic structural features of individual nanocrystals. The bioapatite single crystal was found to crystallize in space groups *P*6_3_ and *P*6_3_/*m*, with lattice parameters a = 9.414 Å; c = 6.881 Å, and showed no evidence of carbonate substitution. The measured Ca/P ratio on single crystals was 1.65 on average.

## Graphical abstract

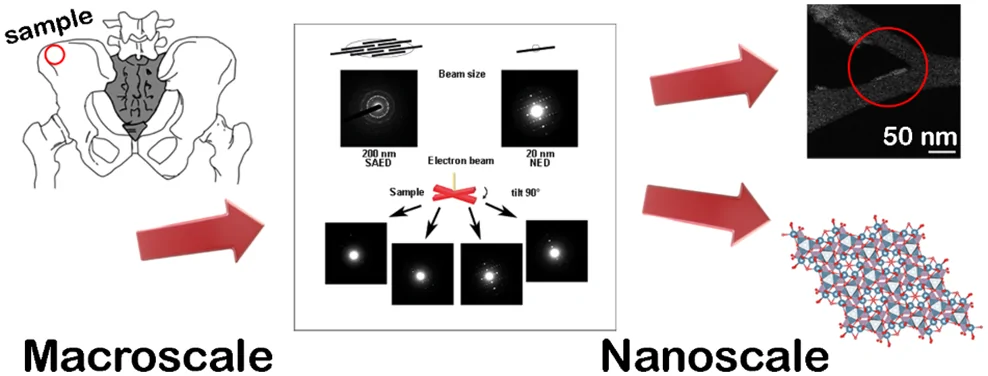

## Introduction

Traditionally, bones are described as a complex heterogeneous biocomposite of organic (mostly collagen) and inorganic components (hydroxyapatite) with several hierarchical levels in texture. A special type of hydroxyapatite is the form most closely approximating the inorganic phase of bones and teeth, it is called bioapatite hereafter in this study. Based on previous studies, bioapatite is considered as composed by “poorly crystallized, CO_3_-substituted hydroxyapatite”.^1–9^

There are multiple possible substitutions in the apatite crystal structure, for instance, on the Ca site (Na, Mg), while CO_3_ substitutions are possible in two crystallographic positions as well, either in OH–CO_3_ (type A) or PO_4_–CO_3_ (type B).^6,10,11^ To maintain the stoichiometry, these substitutions are generally accompanied by associated substitutions. The commonly used approach to distinguishing between type-A CO_3_ and type-B CO_3_ substitutions is a combined application of Fourier transform infrared spectroscopy (FTIR) and X-ray powder diffraction (XRPD).

It is necessary to distinguish between recent and archeological bone specimens. While the constituent mineral phase is bioapatite in both cases, differences may arise due to physicochemical alterations occurring during burial.^12–17^ Differences may also occur between the bioapatites constituting healthy and diseased bone. However, it has not yet been studied whether these differences are manifested at the structural level due to the difficulty in collecting single-crystal diffraction data.

On the basis of numerous studies, there are differences both in chemical composition, shape, crystal size, and biological role, and in these studies, FTIR, XRPD, Raman spectroscopy, scanning electron microscopy (SEM), and transmission electron microscopy (TEM) analyses were mostly performed.^1,11,18–20^ So far, the human bioapatite structure has only been studied in tooth enamel,^19^ and the structure of bioapatite derived from bone has not yet been determined. This is due to the organic material-rich environment and the small nanocrystallite size. FTIR and Raman analyses can be only performed on a few tens of micrometers, so the analysis of objects smaller than this threshold will produce data corresponding to the average volume. In the presence of high noise or polyphase measurements, the experiment may lead to misleading results. XRPD results in a 1D diffractogram of an average volume of the sample, where for small nanocrystallites, peak broadening is often severe and in the presence of multiple phases, the peak overlap may hinder the determination of the correct space group. Also, it is worth mentioning that, according to the literature, the typical size of bone bioapatite is about 50 × 25 × 5 nm in bone, explaining the current challenge in characterizing bioapatite with the above-mentioned techniques, except for TEM.^18,21,22^

In contrast to the above-mentioned techniques, TEM allows the examination of chemical composition and structural properties of materials even at nanometer scale. High-resolution (HRTEM) enables atomic resolution imaging in real space and the examination of local structural defects, however, its high radiation fluence is necessary and can affect small surfaces and beam-sensitive materials, like bioapatite.^1,18,21–23^

Therefore, if the size of the crystal allows it, neutron or X-ray diffraction can be utilized, where mapping the whole 3D reciprocal space by rotating the crystal and/or the detectors, thus achieving the most accurate structural details possible. Nevertheless, standard diffraction techniques are limited by the size of the crystals, where it is extremely challenging to break the nanometer barrier due to optical and instrumental limitations. To overcome these limits and allow for structural characterization of the smallest nanocrystals, 3D electron diffraction (3D ED) techniques can be exploited. 3D ED is often performed in custom TEMs, where high-energy electrons are used to collect single-crystal diffraction data, in an equivalent manner with respect to neutron or X-ray methods. The main advantage of using an electron beam to study small bioapatite nanocrystals is the possibility to use electromagnetic lenses to shape and manipulate the probe size to match the crystal size, down to a few tens of nm.

Case studies using 3D ED to characterize small nanocrystals have already been reported. Structure determination of unknown inorganic minerals has been conducted on materials such as BaSO_4_ crystals about 200 nm in size,^24^ or LaPO_4_ crystals about 25 nm in size embedded in a complex glass ceramic matrix.^25^ Other groups instead focus their research on structural characterization of nanoparticles (NPs) such as very small indium tin oxide (ITO) crystals about 10 nm in size,^26^ pseudobrookite about 30 nm in size,^27^ perovskite materials such as lead chalcohalides NPs about 50 nm in size,^28^ or heterostructure materials like a 35 nm film of PrVO_3_ on a substrate of SrTiO_3_,[!bibref_3] which are mostly materials for energy applications, where researchers are looking for connections between the crystal structure peculiarities and their final application. In all of the cases mentioned above, semiautomated or manual 3D ED data collection has been conducted. This is due to the small size of the crystals investigated increasing the challenge of collecting high-quality single-crystal data from the same volume of sample during the tomography experiment drastically.

There is still much to be discovered in the field of 3D ED, e.g., improving spatial or temporal resolution, faster data collection and processing, and more accurate sample tracking during decision making.^29^ The latter is of critical importance and enables the determination of the structure of materials that are an order of magnitude smaller, reaching the size of single crystals of a few tens of nanometers. Thus, thanks to the latest developments in 3D ED,^30^ it has become possible to determine the actual structure of bone bioapatite. Despite the extensive literature on bone apatite research and the fact that apatite-based biomaterials are a cutting-edge research field, the structural analysis of bioapatite formed in bone has not yet been carried out.3D ED measurements were performed on a single crystal of bioapatite of a few tens of nanometers from a diseased bone sample obtained from an archeological find, resembling one of the smallest nano crystallographic characterizations ever done by a fully automated single-crystal 3D ED approach.

## Materials and Methods

The archeological bone samples were taken from a male individual (inventory number: 2017.4.9. in Hungarian Natural History Museum, Public Collection Center) aged between 40 and 50 years. The individual was excavated from the grave 9/B of Kehida-Fövenyes site (near Kehidakustány, Hungary) dated between the last third of the eighth century AD and first half of the ninth century AD.^31^ The anthropological and paleopathological investigations were carried out by standard osteological methods from a macroscopic approach.^32^ The osteological changes at multiple locations on the skeleton were consistent with secondary neoplastic involvement of the skeletal system.^33^ The sample was taken from the right pelvic bone.

For the TEM study, the sample was gently crushed under ethanol in an agate mortar, and a drop of the suspension was deposited onto a lacey-carbon-covered Cu grid (Ted Pella). Bright-field (BF), HRTEM, and selected-area electron diffraction (SAED) were carried out with a JEOL 2100, equipped with a field emission gun and working at 200 kV accelerating voltage and recorded using a Gatan U1000 CCD camera.

Elemental mapping on selected areas was performed using a JEOL 2200FS microscope in scanning mode (STEM) equipped with an energy-dispersive X-ray spectroscopy (EDS) system (Ultim Max TEM windowless detector, Oxford Instruments plc, UK). The respective maps were recorded at a magnification of 600kX at a resolution of 512 × 512 pixel, resulting in a measured area of 256 nm^2^.

3D ED measurements were carried out with an FEI Tecnai F30 S-Twin TEM equipped with a field emission gun, operating at 300 kV, using a 10 μm condenser aperture, in STEM microprobe mode. Automated Diffraction Tomography (ADT) method was used as data collection protocol and was executed using the PyFast-ADT software in combination with an ASI Timepix1 Hybrid Pixel Detector (HPD) (512 × 512 pixels), at 300 mm camera length and 2s exposure time. In the ADT method, the goniometer was moved iteratively to each probed alpha tilt angle and remained stationary while each pattern was recorded. To integrate the 3D reciprocal space, the ADT method employed a precession movement of the electron probe. Due to the crystal size, the 3D ED data collection was combined with the precalibration crystal tracking technique. A pre-experiment was performed to map the crystal movement across the whole tilt range investigated. Using the PatchworkCC algorithm, a crystal tracking file was automatically generated, recording the xy coordinates of the target at specific tilt angles. Then, the electron beam was shifted accordingly during the 3D ED data collection, minimizing the number of empty frames and the experimental time compared to manual data acquisition.^24^ All 3D ED data collections were performed with a FISCHIONE tomography sample holder. Each Nano Beam Electron Diffraction (NBED) pattern was acquired using a beam size diameter of 20 nm, tilt step 1 deg/image, 90 deg overall tilt range (between −45 and +45 deg), and integrated by a precession movement of the electron probe with a precession angle of 1°, controlled by a DigiSTAR unit (Nanomegas SPRL), as described by.^30^

The PETS software package was used for the 3D ED data processing. Crystal structure determination was performed by SIR2014 software,^34^ and the structure was subsequently refined using JANA2020 software, taking both kinematical and dynamical scattering into account.^35,36^

## Results

BF images were used to show the size and elongated needle shape of bioapatite (Figure 1a). Based on the BF images, the biggest crystallite sizes were measured to be 107*11 nm, where the average crystal length is 52 nm, width is 7.4 nm, medians are 49 and 7.4 nm, with respective standard deviations 18.8 and 1.7 nm (Appendix Figure S1a and 1b, Figure S2). HRTEM imaging was performed to visualize the crystal structure and morphology of individual bioapatite crystallites at high resolution (Figure 1b and 1c). Some crystal viewed close to the [001] direction (Figure 1b), some other was observed along the [1̅02̅] direction, confirming the elongation of bioapatite crystallites was parallel to [010] (Figure 1c). Fast Fourier- Transform (FFT) analysis of HRTEM image shown in Figure 1b may suggest the hexagonal symmetry of the crystal lattice. This indicates the highest possible symmetry of p6 in projection, which does not exclude the possibility of lower symmetry in three dimensions. No structural disorder was observed at the grain boundaries of the crystals. This is particularly important because the observed crystals are only a few unit cells wide, demonstrating that these are small, well-ordered nanocrystallites rather than poorly crystalline, randomly oriented crystallites. However, these HRTEM images do not allow for more detailed structural observations.

**Figure 1:**
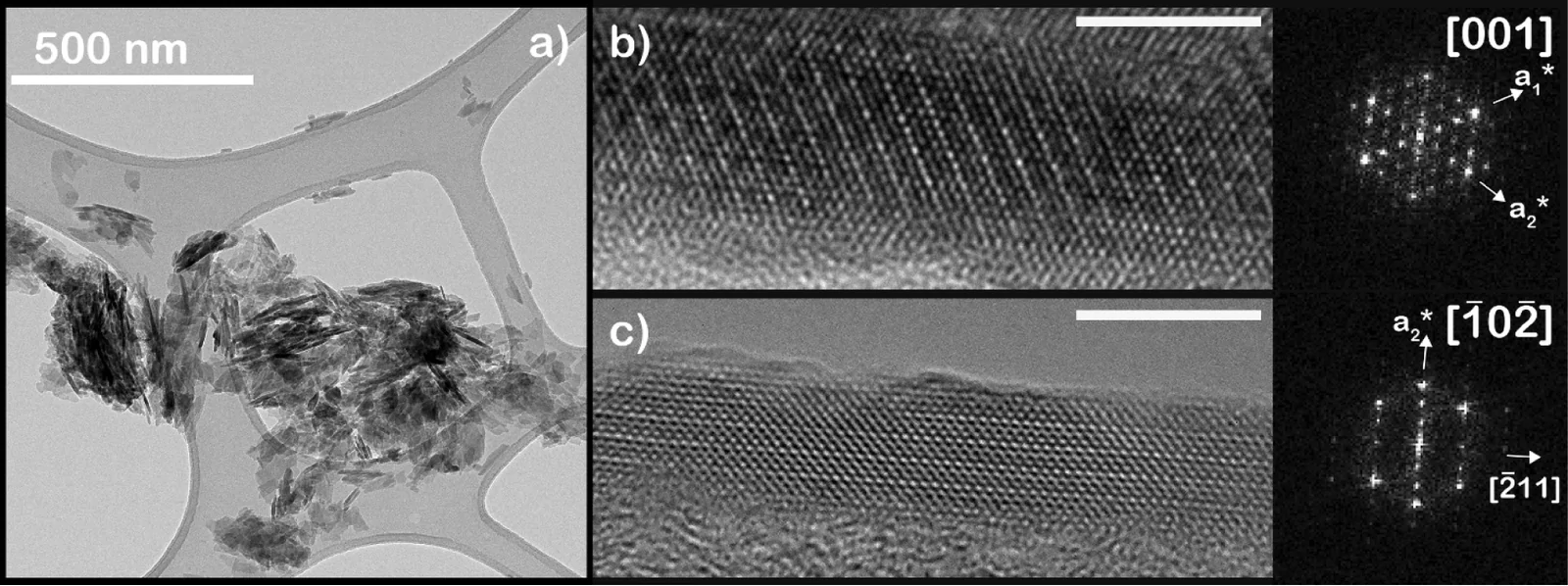
a) TEM BF image about the sample b) HRTEM images show in close to zone [001] and c) [1̅02̅] and their FFT that crystallites are well crystallized, no disorder is present. Scale bar is 5 nm on HRTEM images.

STEM-EDS mapping was carried out on the crystallite clusters to measure their chemical compositions. On the basis of the elemental maps collected, Ca and P are present as major elements, alongside Mg and Na as minor elements. Ideally, in apatite–OH, the Ca/P ratio should be 1.67, which may vary because of elemental substitution. Substitution may occur at the Ca position (Mg and Na) or at the PO_4_ position (CO_3_). During element mapping, a few anomalies in Ca/P values were found, which would indicate a high Ca content unrealistic in any known CaPO_4_ phase, while in most of the cases a Ca/P ratio of 1.65 ± 0.01 was found (Appendix Table S1). If chemical compositions are examined according to single crystal data points, the above-mentioned outliers can be clearly identified as impurities. Similarly, the XRPD data indicate a considerable amount of calcite crystals in the sample (above 12 wt %, Appendix Figure S2), however, at the nanoscale, it is possible to find calcite crystals alongside bioapatite single crystals, thus influencing the average Ca/P ratio of the maps collected. However, the Ca/P ratio of bioapatite single crystals is within the margin of error for the theoretical Ca/P ratio. Therefore, it can be concluded that there is no CO_3_ substitution present in the structure at the PO_4_ position.

Initial attempts to obtain single-crystal diffraction patterns using conventional SAED were unsuccessful. Even with the smallest available SA aperture, which limits the examined area to approximately 250 nm, the diffraction patterns showed rings characteristic of polycrystalline apatite (Appendix Figure S1c and 1d). These results reflect the fact that multiple bioapatite nanocrystals were present within the selected area because of their small individual dimensions.

To obtain single-crystal diffraction data from individual bioapatite crystals, NBED mode had to be employed, and a beam size diameter of 20 nm was used to increase the chances to gather single-crystal diffraction data using 3D ED.

However, when such a small electron probe is used to collect 3D ED in a conventional TEM, instrumental instabilities such as goniometer misalignments and unreproducible shifts of the sample during the experiment may occur, and keeping the crystal under the electron probe for the whole experiment becomes challenging. Usually, the solution is to manually collect every single diffraction pattern of the data set, which is a long procedure prone to errors. Instead of this, the 3D ED data set was collected using a fully automated crystal tracking method implemented in the PyFast-ADT software, which is specifically designed to tackle these issues, and enabled the automated data collection of the smallest nanocrystals shown in this work.^30^

Despite the challenging size range, 3D ED single crystal data were successfully collected in a fully automated way, from a single bioapatite nanocrystal of ∼ 7 nm size in its shortest dimension (Figure 2). The 3D reciprocal space reconstruction enabled *ab initio* structure solution and both kinematical and dynamical refinement of the crystal structure. This represents the first successful structure determination of bone bioapatite at the single crystal level.

**Figure 2:**
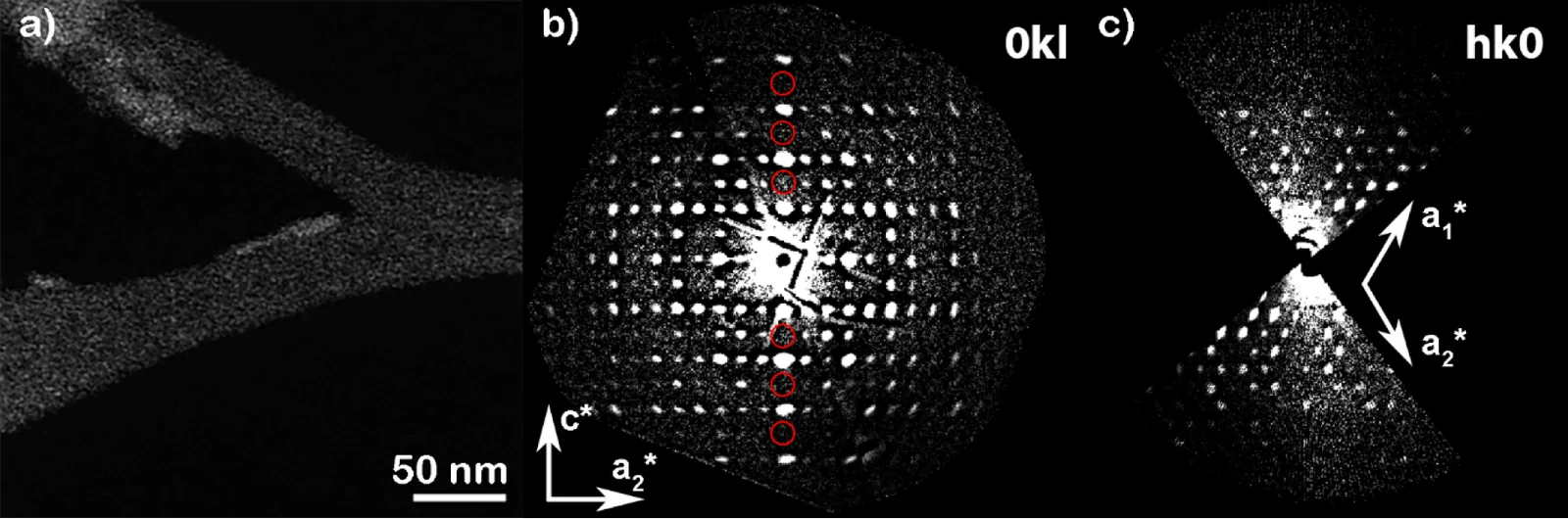
a) A STEM image of the measured bioapatite crystallite b) 3DED sections in 0kl and c), hk0. 00l = 2+n extinction is visible along c*. No evidence of any structural disorder is present.

The structure was initially solved using the SIR2014 software package. Subsequently, it was refined using JANA2020, which accounts for both kinematical and dynamical scattering effects, which is critical for electron diffraction data. Two possible space groups within the hexagonal system were tested: the centrosymmetric *P*6_3_ */m* and the noncentrosymmetric *P*6_3_. Both belong to the same Laue group (6/m), and that is the reason why they cannot be reliably distinguished by powder diffraction methods due to systematic absences. The refinement statistics and the refined crystallographic parameters are presented in Tables 1 and 2, respectively. The lattice parameters are a = 9.414Å and c = 6.881Å with α=β=90° and γ=120°, confirming the hexagonal crystal system. These values are consistent across both space group refinements.

**Table 1:** Statistical Parameters of Dynamical Structure Refinement in P6_3_ and P6_3_/m

Space group	R(obs)	wR(obs)	R(all)	wR(all)	GOF(obs)	GOF(all)
***P6***_***3***_	13.51	15.25	28.71	16.06	5.18	2.45
***P6***_***3***_ ***/m***	13.60	15.87	27.37	16.68	5.38	2.54

**Table 2:** Lattice Parameters and Atom Coordinates in P6_3_ and P6_3_/m Space Groups

a	b	c	α	β	γ	a	b	c	α	β	γ
9.414	9.414	6.881	90	90	120	9.414	9.414	6.881	90	90	120
***P6***_***3***_						***P6***_***3***_ ***/m***					
**Atom**	**x**	**y**	**z**	**Ueq/Uiso**	**Occ**	**Atom**	**x**	**y**	**z**	**Ueq/Uiso**	**Occ**
**Ca**	0.3333	0.6667	0.5172	0.015	1	**Ca**	0.3333	0.6667	0.5024	0.011	1
**Ca**	0.3333	0.6667	1.0141	0.015	1	**Ca**	0.0083	0.2535	0.7500	0.019	1
**Ca**	0.7467	0.7556	0.7709	0.022	1	**P**	0.3980	0.3720	0.7500	0.018	1
**P**	0.6008	0.6275	0.2612	0.019	1	**O**	0.3262	0.4845	0.7500	0.024	1
**O**	0.6638	0.7537	1.0918	0.021	1	**O**	0.0833	0.3517	0.4323	0.033	1
**O**	0.5167	0.8441	0.7563	0.024	1	**O**	0.5874	0.4661	0.7500	0.037	1
**O**	0.6342	0.7087	0.4562	0.029	1	**O**	0.0000	1.0000	0.6667	0.045	0.5
**O**	0.5358	0.1212	0.7355	0.022	1	**H**	0.0000	1.0000	0.5140	0.054	0.5
**O**	0.0000	1.0000	0.8468	0.047	1						
**H**	0.0000	1.0000	0.9994	0.057	1						

The dynamical refinement in *P*6_3_/*m* yielded R­(obs)=13.60%, wR­(obs)=15.87%, and GOF (obs)=5.38. The dynamical refinement in *P*6_3_ showed similar R-values with R­(obs)=13.51%, wR­(obs)=15.25%, and GOF (obs)=5.18. Both refinements converged successfully with all data, yielding R­(all) values of 27.37% and 28.71% for *P*6_3_/*m* and *P*6_3_, respectively. Therefore, only on the basis of the R-value statistics, it is not possible to discern between the two space groups.

There are a few minor crystallographic differences between the two models, mainly due to the greater degree of freedom allowed in the lower *P*6_3_ space group, which does not contain the center of inversion present in *P*6_3_/*m*. In the *P*6_3_/*m* model, Ca occupies two distinct positions, whereas in the *P*6_3_ model, it occupies three positions. However, the observed electron density map shows well-defined atomic positions, while the difference map does not contain a meaningful additional residual potential in both space groups. The atomic displacement parameters are reasonable for all of the atoms in both models, resembling a well-ordered structure.

The H atom located at the channel-filling OH position was also successfully determined *ab initio* after dynamical refinement. OH position exhibits a slight displacement along the *z*-direction in space group *P*6_3_, whereas in the case of *P*6_3_/*m*, this displacement is averaged out due to the mirror plane (Appendix Figure S3), and the occupancy is correspondingly halved. Considering a single unit cell, the H may point either upward (z+) or downward (z-). Since diffraction data represent the average structure of a given volume—potentially encompassing several million unit cells—their reliability depends on the degree of structural order. If a systematic displacement exists at a given atomic position, this will manifest in the diffraction pattern via the structure factor. When such displacement occurs frequently, indicating a disordered structure, diffuse scattering appears. In the case of bioapatite, no diffuse scattering is observed, the only atoms exhibiting positional “looseness” are O and H at the channel-filling OH position. Since the number of unit cells constituting the crystallite is finite, it is possible for the H atom to point upward (z+) in one unit cell and downward (z-) in another. Following structure refinement, the *P*6_3_/*m* space group is reflecting the averaging of these two H atoms positions, since the channel-filling site blends the two orientations together. Refinement in the *P*6_3_ space group, by contrast, allowed the H atom position to be well determined, with no residual potential remaining. In this case, however, all H atoms within a given unit cell are constrained to point in the same direction. At the same time, it should be emphasized that the difference between the two models concerns nothing more than the precise position of a single H atom, in a volume that is not bigger than approximately 2000 unit cells. This is such a low scattering power that explains the similar statistical results obtained for the two refined space group models.

## Discussion

A complex transmission electron microscopy study was performed on a single crystal of bioapatite from a tumorous archeological bone sample, thus providing a complete picture of its structure and chemical composition. The hexagonal structure is confirmed by HRTEM and 3D ED data, with measured angles and lattice parameters consistent with the hexagonal system. Notably, the measured average Ca/P ratio of 1.65±0.01 of individual bioapatite crystals is within the margin of error of the theoretical Ca/P ratio of 1.67 for stoichiometric hydroxyapatite. The result established in this recent study, combined with the structural refinement results, indicates the absence of CO_3_ substitution in the PO_4_ position (type-B substitution), contrary to the widely accepted characterization of bioapatite as “poorly crystalline, CO_3_-substituted hydroxyapatite”: The lack of observable type-A substitution (CO_3_ replacing OH in the channels) is consistent with the structural data as well, as the OH site shows full occupancy with reasonable displacement parameters.

The refinement results show that both *P*6_3_ and *P*6_3_/*m* represent valid structural solutions. However, the choice between these two space groups must consider not only statistical parameters but also physical and structural arguments. The key structural difference lies in the symmetry operation: *P*6_3_/*m* contains a mirror plane perpendicular to the *c*-axis at z = 0.75, which constrains the OH groups in the channels to be perfectly aligned along the *c*-axis. In contrast, *P6*_3_ allows for tilting or disorder of the OH columns due to the absence of this mirror plane constraint.

Understanding the relationship between tumor formation and bioapatite crystallization could have significant implications for diagnostic and preventive applications. If pathological bone formation produces characteristic structural signatures, then these could potentially serve as markers for disease progression or treatment response. Given the uncertainties of the archeological sample, it is essential to repeat this investigation using recent bone samples from both healthy and pathological sources.

## Conclusion

This research demonstrates that 3D ED can successfully acquire single-crystal diffraction data fully automatically from crystals of a few tens of nanometers size, enabling the determination of their structure, which is extremely challenging to measure with every other kind of investigation technique currently available. The methodology established here provides a foundation for systematic studies of bioapatite formation under various physiological and pathological conditions, which may ultimately contribute to an improved understanding and prevention of bone diseases and the development of new biomaterials.

Both *P*6_3_ and *P*6_3_/*m* models constitute acceptable structural solutions. The distinction between *P*6_3_ and *P*6_3_/*m*, although resting on the position of a single H atom, carries significance beyond crystallographic formalism. *P*6_3_/*m* averages the OH proton over two symmetry-equivalent orientations via its mirror plane, implying disorder, whereas *P*6_3_ permits local ordering of proton position within individual unit cells—potentially reflecting the tightly controlled, protein-templated environment of bioapatite nucleation and extending biological control down to the atomic scale of channel occupancy. It is worth noting, however, that distinguishing genuine local ordering from an artifact of finite crystallite averaging will require complementary techniques, such as solid-state NMR. Although the sample analyzed here derives from an archeological individual, this study nonetheless demonstrates, as a proof of concept, that atomic-resolution structural characterization of such beam-sensitive, nanometric crystallites is now achievable, opening the door to similar analyses on recent, nonarcheological, healthy, and diseased bone materials in future studies. The challenge of determining structures from such small crystallites, consisting of only a few thousand atoms, represents a significant achievement in the field of nanocrystallography and demonstrates the power of modern 3D ED techniques for studying biological materials at the nanoscale.

## Supplementary Material







## Notes

(CIF)
(CIF)
Additional experimental details include TEM BF and SAED patterns (Figure S1), XRD measurements (Figure S2), a structural model (Figure S3), and a STEM-EDS data set (Table S1) (PDF)
