# Supplementary material for: Structure Determination of a Single Bioapatite Crystal from an Archeological Metastatic Cancer Bone – a TEM Case Study
Source: ACS Omega. 2026 Oct 12;11(42):64737–43. doi: 10.1021/acsomega.6c05043 (PMC13717791; doi:10.1021/acsomega.6c05043)
Supplement: Supplementary file 1 [file ao6c05043_si_003.pdf]

Appendix file

Structure determination of a single bioapatite crystal from an archaeological metastatic cancer bone – a TEM case study

Zsolt Dallos [a*], Marco Santucci [b], Krisztián Kiss [c,d,e,f], Melanie Bauer [b], Michael Burtscher [g], Zoltán Kovács [h], Tamás Szeniczey [c], Antónia Marcsik [i], Erika Molnár [i], Krisztina Buczkó [j,k], Daniel Kiener [g], Ute Kolb [b,l*], Tamás Hajdu [c,m*], István Dódony [n]

a Institute of Geoscience, Friedrich Schiller University Jena, Jena, D-07743, Germany
b Institute of Inorganic Chemistry and Analytical Chemistry, Johannes-Gutenberg-University, Mainz, Mainz, D-55128, Germany
c Department of Biological Anthropology, Eötvös Loránd University, Budapest, H-1117, Hungary
d Institute of Archaeogenomics, ELTE Research Centre for the Humanities, Budapest, H-1097, Hungary
e Institute of Practical Methodology and Diagnostics, Faculty of Health Sciences, University of Miskolc, Miskolc, H-3515, Hungary
f Lendület "Momentum" Bioarchaeology Research Group, MTA–ELTE HTK, Budapest, H-1097, Hungary
g Department Materials Science, Technical University of Leoben, Leoben, A-8700, Austria
h HUN-REN Centre for Energy Research, Budapest, H-1121, Hungary
i Department of Biological Anthropology, University of Szeged, Szeged, H-6726, Hungary
j Center for Natural Sciences, Limnology Research Group, University of Pannonia, Veszprém, H-8200, Hungary
k Department of Botany, Hungarian Natural History Museum, Budapest, Hungarian National Museum Public Collection Centre, Budapest, H-1431, Hungary
l Institute for Applied Geosciences,Technical University of Darmstadt, Darmstadt, D-64287, Germany
m Centre for Applied Bioanthropology, Institute for Anthropological Research, Zagreb, Croatia
n NANOLAB, Research Institute of Biomolecular and Chemical Engineering, University of Pannonia, Veszprém, H-8201, Hungary

*Email: zsolt.dallos@uni-jena.de

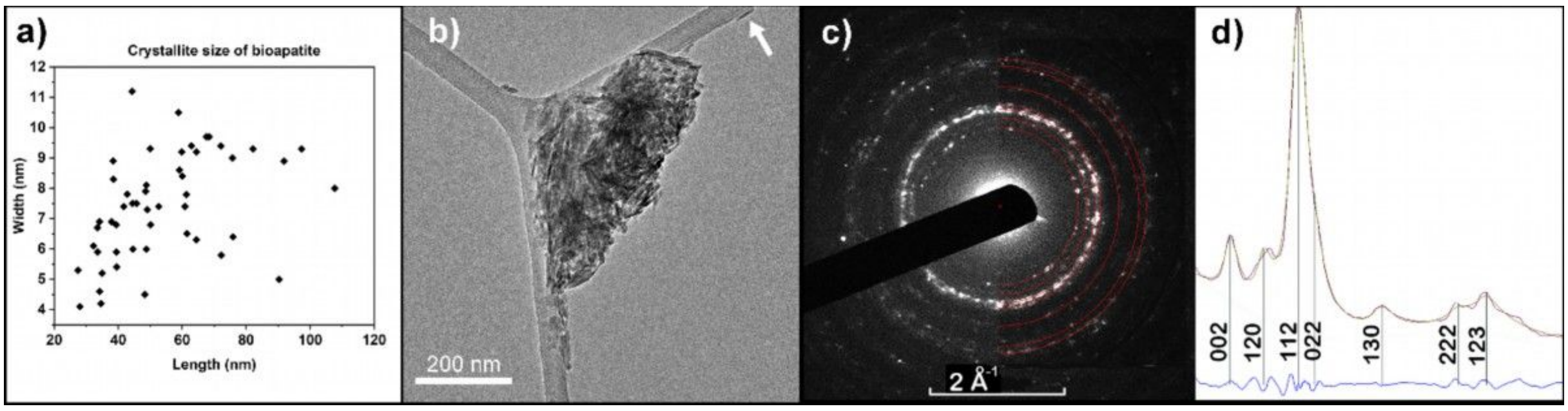

Figure S1 a) Particle size distribution, b) TEM BF image and c) SAED of the bone bioapatite. d) Intensity profile of the intensities displayed on the SAED, indexed by apatite hkl values.

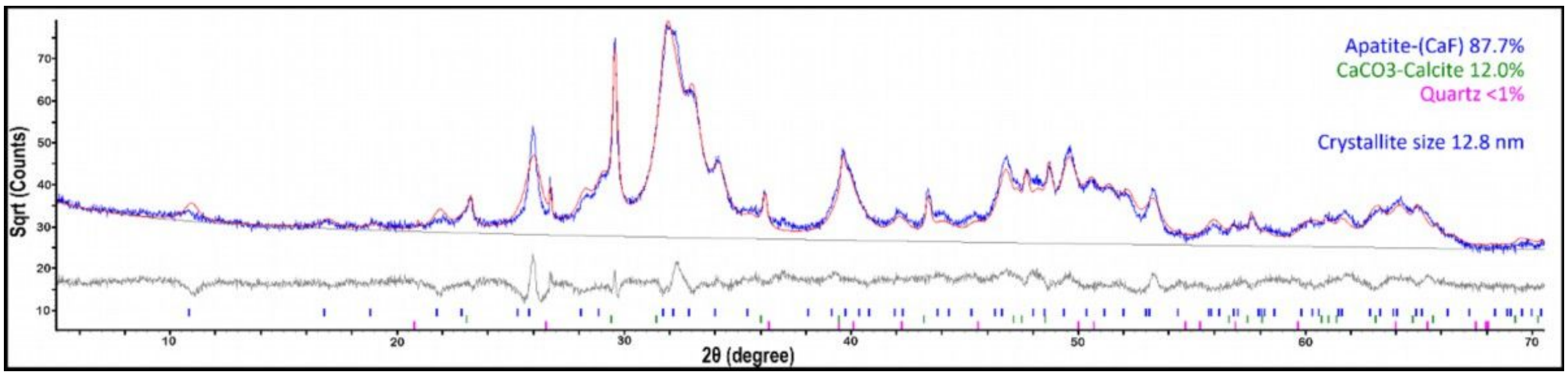

Figure S2 XRD diffractogram of the bone sample from Kehidafövenyes.

Table S1 Chemical composition of individual crystals calculated from the STEM-EDS map.

Spectrum Label	O	Na	Mg	P	Ca	Ca/P
Spectrum 1	81.21	0.37	0.42	7.31	10.69	1.46

Spectrum 2	80.00	0.39	0.66	6.77	12.17	1.80
Spectrum 3	80.56	0.00	0.00	7.74	11.70	1.51
Spectrum 4	83.10	0.39	0.00	6.42	10.09	1.57
Spectrum 5	80.92	0.00	0.71	6.74	11.64	1.73
Spectrum 6	78.15	0.21	0.38	8.59	12.70	1.48
Spectrum 7	78.99	0.34	0.20	8.11	12.35	1.52
Spectrum 8	82.63	0.00	0.95	6.29	10.14	1.61
Spectrum 9	82.46	0.00	0.00	6.65	10.89	1.64
Spectrum 10	84.17	0.00	0.00	5.96	9.86	1.65
Spectrum 11	83.07	0.00	1.00	6.02	9.90	1.65
Spectrum 12	79.46	0.00	0.32	7.14	13.07	1.83
Spectrum 13	80.12	0.00	0.55	7.07	12.26	1.73
Spectrum 14	80.71	0.00	0.00	7.31	11.97	1.64
Spectrum 15	79.32	0.00	0.18	7.08	13.41	1.89
Spectrum 16	79.36	0.00	0.23	7.60	12.81	1.69
Spectrum 17	80.32	0.00	0.48	7.14	12.06	1.69
Spectrum 18	78.48	0.00	0.00	8.74	12.78	1.46
Spectrum 19	78.92	0.00	0.00	7.64	13.42	1.76
Spectrum 20	79.19	0.00	0.19	8.18	12.46	1.52
Spectrum 21	79.79	0.17	0.22	7.11	12.71	1.79
**Average:**	80.52	0.09	0.31	7.22	11.86	1.65
**Mean:**	80.12	0.00	0.22	7.14	12.17	1.65
**Standard deviation:**	1.68	0.15	0.32	0.77	1.16	0.13

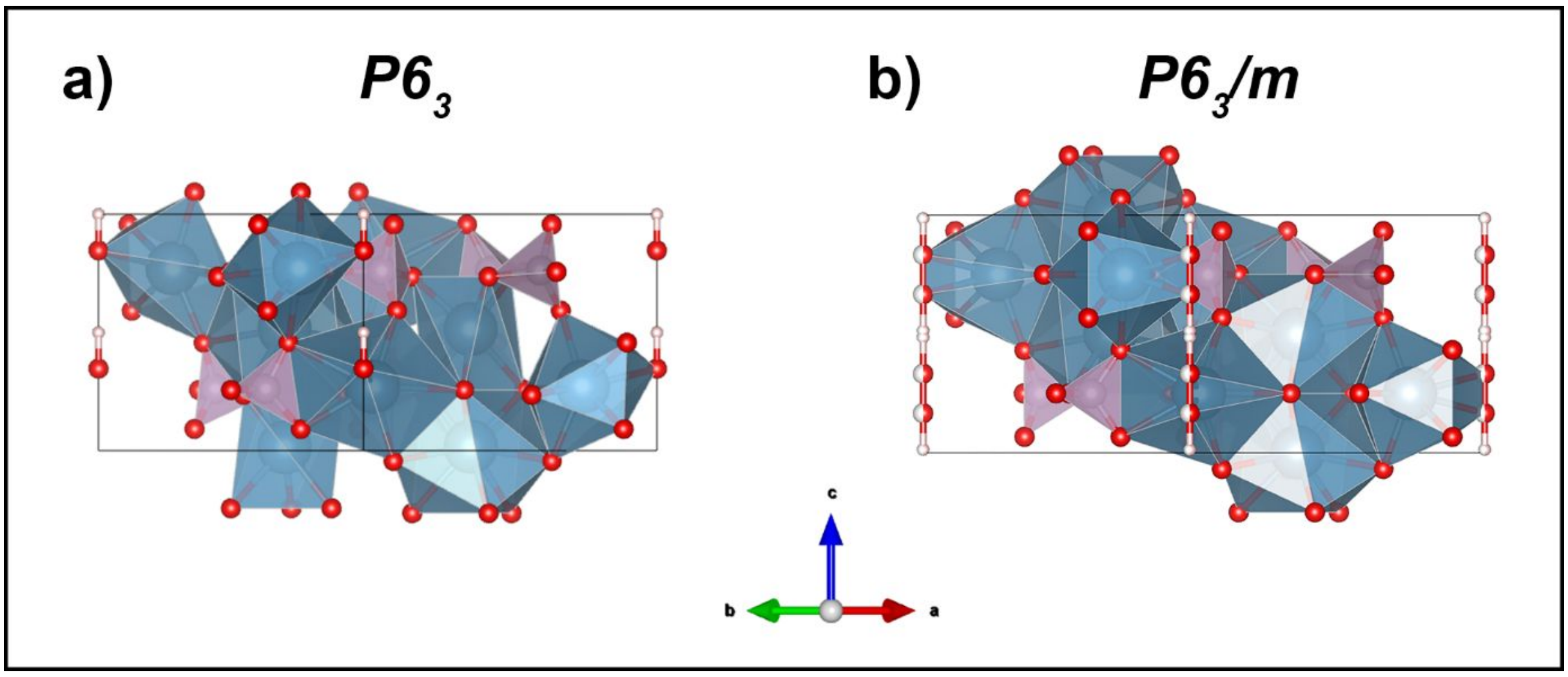

Figure S3 Structural model for the space group a) $P6_3$ and b) $P6_3/m$.
